# Scabies crustosa in a recipient of an allogeneic stem cell transplantation

**DOI:** 10.1007/s15010-020-01422-7

**Published:** 2020-04-08

**Authors:** Maximilian Christopeit, Dominic Wichmann

**Affiliations:** 1 Department of Stem Cell Transplantation, University Medical Center Hamburg-Eppendorf, Martinistrasse 52, 20246 Hamburg, Germany; 2 Department of Intensive Care Medicine, University Medical Center Hamburg-Eppendorf, Martinistrasse 52, 20246 Hamburg, Germany

**Fig. 1:**
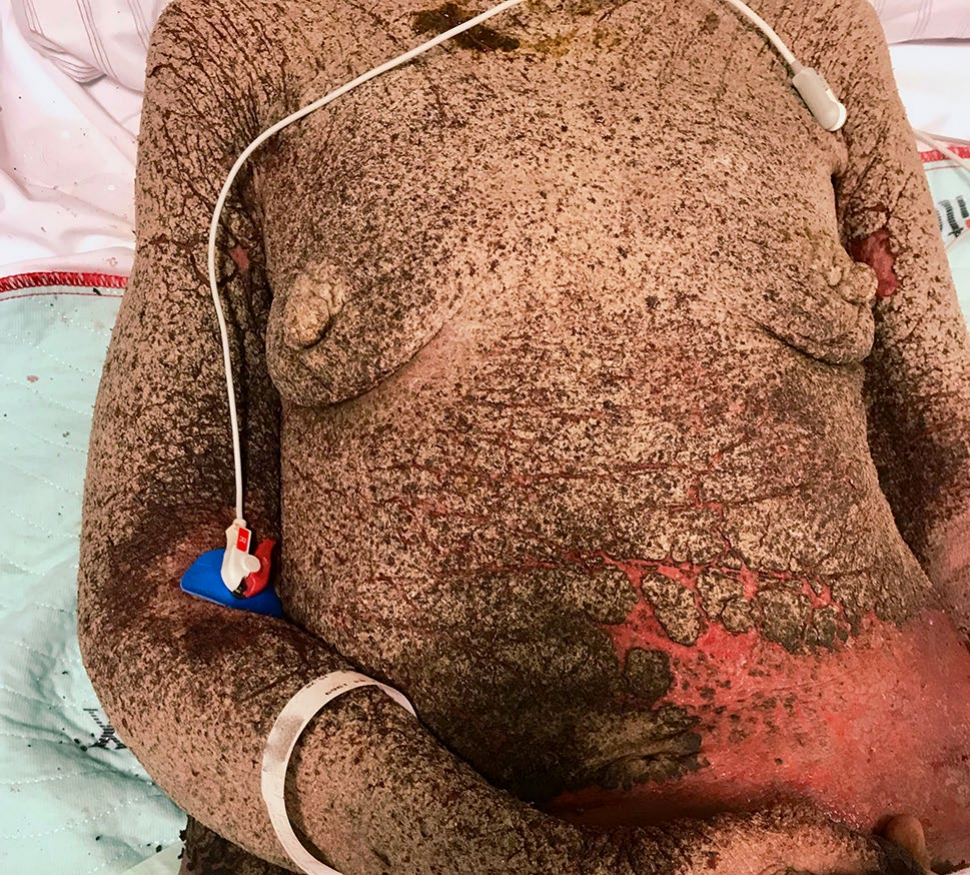
A 49-year-old female patient after allogeneic hematopoietic stem cell transplantation for Hodgkin’s lymphoma with classical signs of crusted scabies

A 49-year-old woman presented with crustuous efflorescences covering ≈ 70% of an erythematous skin (Fig. 1). She developed hypotension and a partially compensated metabolic acidosis (pH: 7.16;* p*CO_2_: 32 mmHg).

Allogeneic hematopoietic stem cell transplantation from a matched unrelated donor following myeloablative conditioning including anti-T-lymphocyteglobulin had been performed 2 months prior to treat relapsed stage IVB Hodgkin’s lymphoma. A localized scabies infection occurring at the end of the aplastic phase was treated with ivermectin in the recommended dose of 200 µg/kg bodyweight on days 1 and 8, each. After transplantation, visits to the outpatient unit frequently deviated from schedule, clinical status was poor, and cyclosporine levels were below target level.

Scabies crustosa, historically referred to as scabies norvegica, and septic shock were diagnosed. Despite fluid and vasopressor resuscitation, antimicrobial, anti-parasitic and anti-inflammatory drug treatment, continuous renal replacement therapy, and respiratory support, she died due to multi-organ failure 1 day after initial presentation. Blood cultures taken the day before grew *Enterococcus faecium*.

Scabies is a parasitic disease caused by the mite *Sarcoptes scabiei* var. *hominis* [1]. In Western Europe, scabies can be of concern in communities with social problems, and in patients with risk factors such as promiscuity and advanced age. Due to defective T cell responses, immunosuppressed patients are at increased risk of experiencing crusted scabies, previously referred to as scabies norvegica [2, 3]. Production of complement inhibitors by the mites results in an increased risk for secondary infections caused by streptococci and staphylococci in the affected patients [4]. Treatment options for scabies are systemic oral ivermectin (USA: FDA off-label, EU: EMA approved), or topical permethrin [5].
